# Top-down influences on ambiguous perception: the role of stable and transient states of the observer

**DOI:** 10.3389/fnhum.2014.00979

**Published:** 2014-12-08

**Authors:** Lisa Scocchia, Matteo Valsecchi, Jochen Triesch

**Affiliations:** ^1^Milan Center for Neuroscience, Department of Psychology, University of Milano-BicoccaMilan, Italy; ^2^Department of Psychology, Giessen UniversityGiessen, Germany; ^3^Frankfurt Institute for Advanced Studies, Johann Wolfgang Goethe UniversityFrankfurt am Main, Germany

**Keywords:** ambiguous perception, bistability, binocular rivalry, reversible figures, ambiguous structure-from-motion (SFM), top-down control, modeling

## Abstract

The world as it appears to the viewer is the result of a complex process of inference performed by the brain. The validity of this apparently counter-intuitive assertion becomes evident whenever we face noisy, feeble or ambiguous visual stimulation: in these conditions, the state of the observer may play a decisive role in determining what is currently perceived. On this background, ambiguous perception and its amenability to top-down influences can be employed as an empirical paradigm to explore the principles of perception. Here we offer an overview of both classical and recent contributions on how stable and transient states of the observer can impact ambiguous perception. As to the influence of the stable states of the observer, we show that what is currently perceived can be influenced (1) by cognitive and affective aspects, such as meaning, prior knowledge, motivation, and emotional content and (2) by individual differences, such as gender, handedness, genetic inheritance, clinical conditions, and personality traits and by (3) learning and conditioning. As to the impact of transient states of the observer, we outline the effects of (4) attention and (5) voluntary control, which have attracted much empirical work along the history of ambiguous perception. In the huge literature on the topic we trace a difference between the observer's ability to control dominance (i.e., the maintenance of a specific percept in visual awareness) and reversal rate (i.e., the switching between two alternative percepts). Other transient states of the observer that have more recently drawn researchers' attention regard (6) the effects of imagery and visual working memory. (7) Furthermore, we describe the transient effects of prior history of perceptual dominance. (8) Finally, we address the currently available computational models of ambiguous perception and how they can take into account the crucial share played by the state of the observer in perceiving ambiguous displays.

## Introduction

**Figure 1:**
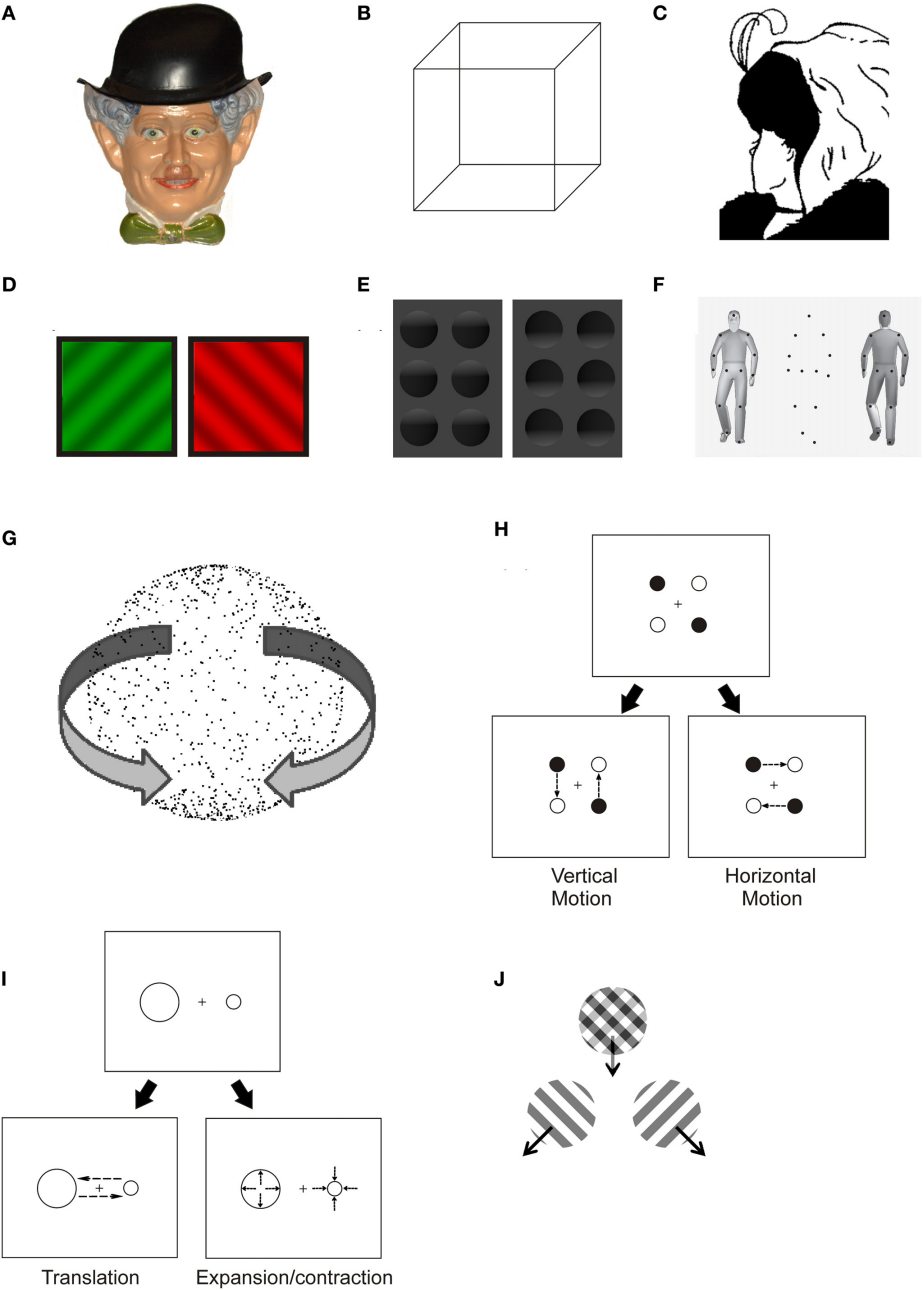
**Examples of ambiguous stimuli**. **(A)** Hollow-face illusion: a concave mask looks like a convex face. **(B)** It is not possible to state which side of the Necker Cube is facing the observer: perspective-based reversals occur between two possible configurations of the cube. **(C)** Hill's Wife/Mother-in-law ambiguous figure: the image can be alternatively perceived as a young or as an old lady. **(D)** Binocular rivalry: two different monocular images are displayed to each eye, causing subjective perception to alternate between the two. **(E)** Shape from shading: under the implicit assumption that light comes from above in a scene, the shading pattern becomes responsible for the perception of convexity or concavity of a visual object. **(F)** The ambiguous point-light walker is a particular instance of structure-from-motion (see **G**): when motion information is added to the display, the dots are perceived as a walker facing toward or away from the observer. **(G)** Static depiction of an ambiguously rotating structure-from-motion sphere: when motion information is added to the display, the stimulus is seen as a three-dimensional sphere and opposite directions of motion alternate in perception. **(H,I)** Two instances of ambiguous apparent motion: in the bistable motion quartet **(H)**, single dots displayed in succession at a fixed frame rate induce the impression of vertical or horizontal translation. In **(I)**, rather than perceiving two static dots of different size being flashed at different positions on the screen, the viewer perceives two dots exchanging positions (translation) or two dots expanding and contracting at two separate locations. **(J)** When two drifting sinusoidal gratings are superimposed, they can be perceived as either a coherently moving plaid pattern or as two semi-transparent gratings moving on top of each other in different directions.

When a physical stimulus does not have a univocal perceptual correspondence we may face two different kinds of illusory experiences: first, the stimulus may be given a “mistaken” perceptual interpretation, thus leading to stable illusions (as in the case of the hollow-face illusion, where a concave mask is steadily perceived as a convex face, Figure 1A). Second, mutually exclusive percepts may alternate under the viewer's eye: a phenomenon called bistable or multistable perception. Strictly speaking, bistable perception is triggered by a physical stimulus that allows for only two alternative interpretations, whereas multistable perception is observed in case of multiple possible alternatives (e.g., Kubovy, 1994). However, the two terms are often used synonymously in the literature. Bistable perception comprises a wide range of illusory phenomena: from reversible figures, where the stimulus configuration enables multiple interpretations (e.g., Figures 1B,C), to binocular rivalry (e.g., Figure 1D), where conflicting monocular images are displayed separately to the two eyes.

**Figure 2:**
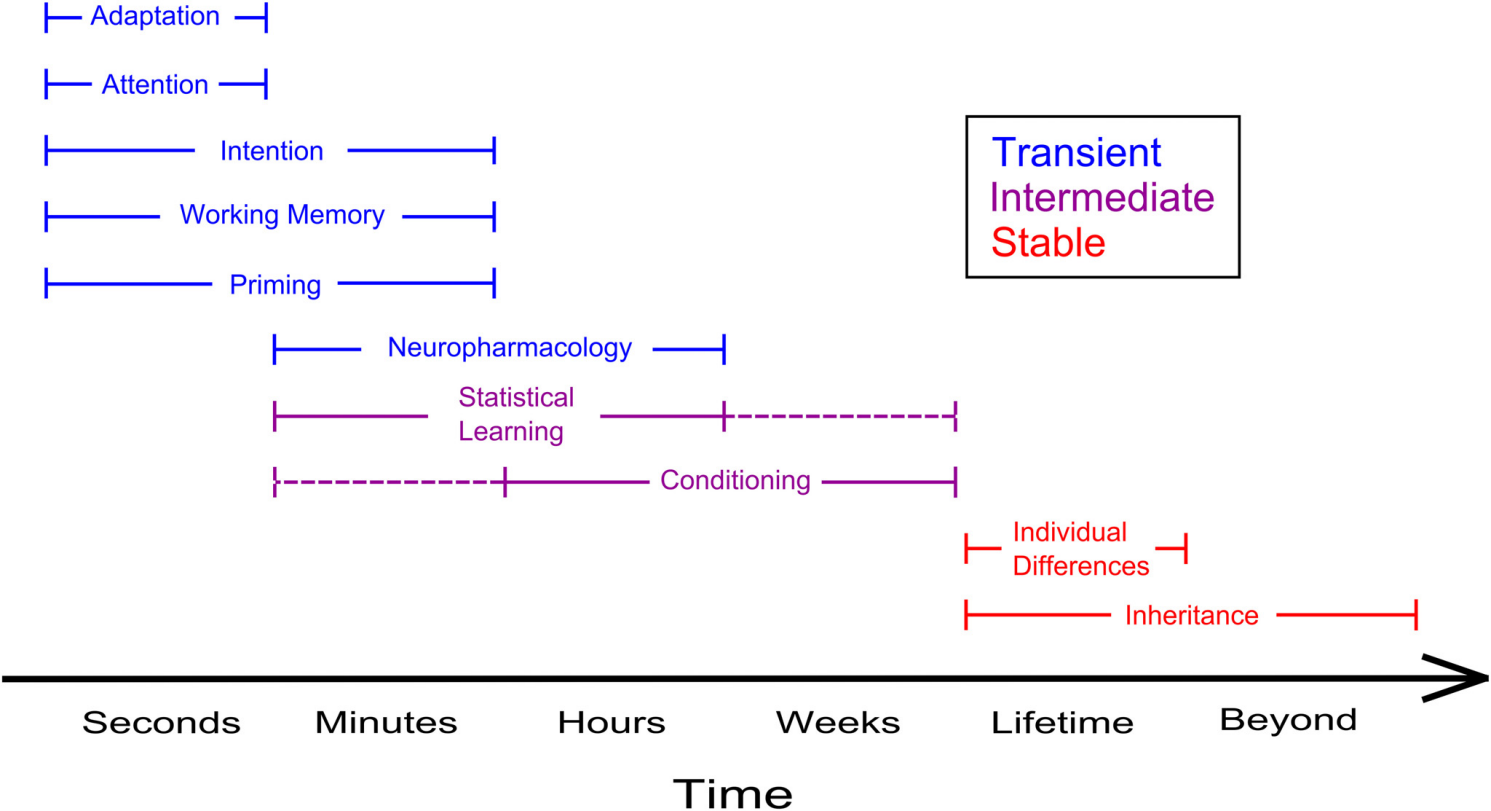
**Summary of the timeframes in which a few top-down determinants of ambiguous perception can be observed**. Phenomena such as adaptation and attention typically dissipate within a window of a few seconds whereas the effects of inheritable individual differences linger on beyond the lifespan of a single individual. Blue and red lines illustrate, respectively transient and stable influences of the state of the observer on ambiguous perception, violet lines designate effects that occur at an intermediate level. Dashed lines indicate timescales under which the effects are plausibly taking place, but that have not been investigated yet.

Although the neural substrata underpinning different kinds of ambiguous perception may be rather diverse, bistable perception poses intriguing questions as to the issue of visual awareness, *in primis* as to the degree to which we can control our conscious visual experience. Top-down influences on ambiguous perception, however, can be exerted at different levels of perceptual processing and across different timescales: for instance, individual differences determined by the genes likely act at a physiological level and their effect persists through a lifetime (and beyond). Instead, the effects related to the voluntary deployment of attention likely act at a higher level of processing and are of a more fleeting nature (Figure 2). Therefore, in the bulk of studies devoted to the topic, we find it useful to trace a distinction between research on how stable and transient states of the observer may affect the phenomenal appearance of bistable objects. Stable states of the observer include manifold aspects, ranging from the effects of prior knowledge and motivation to those of individual and genetic differences. Instead, transient manipulations of the state of the observer include the effects of intention, imagery and working memory, which are achieved via the exertion of an explicit cognitive effort, and the effects of adaptation and prior history of perceptual dominance, which are independent of the observer's voluntary control. The influence of stable and of transient states on ambiguous perception will be covered, respectively in Section Stable States of the Observer and Ambiguous Perception and Transient States of the Observer and Ambiguous Perception.

As ambiguous displays have been employed as a paradigm to investigate perception, and in particular the interplay between the brain and the outside world in determining what is seen, several theoretical models have been put forward to promote our understanding of the determinants of perceptual bistability. Section Modeling Ambiguous Perception: Toward Accounting for the Impact of Stable and Transient States of the Observer will be devoted to the theoretical advances in explaining the mechanisms of top-down modulation of bistable perception, taking into account the role of both stable and transient states of the observer.

## Stable states of the observer and ambiguous perception

### Cognitive and affective aspects in the interpretation of ambiguous displays

The way humans perceive ambiguous stimuli can tell us a great deal about what the visual system “expects” to see and what it “likes” to see. Evolution and life-long learning build into our nervous system knowledge about the world, both explicit and implicit. This knowledge is used to disambiguate stimuli which would otherwise be ambiguous. Furthermore, the motivational value and emotional relevance of a stimulus' interpretation can determine how likely it is to dominate when multiple mutually exclusive percepts are possible. Perhaps the most striking example of a situation where our visual system's understanding of the world imposes an interpretation of a stimulus which is physically ambiguous is the hollow-face illusion (Gregory, 1970). The top-down influence in this illusion is so strong that the hollow-face is not bistable and invariably appears convex when the stimulation is physically ambiguous (i.e., when viewed statically and monocularly under diffuse illumination). Furthermore, the percept of convexity is resistant to contrary sensory evidence from motion parallax, binocular disparity and shading to a great extent. The hollow-face illusion is intriguing also because it clarifies that the semantic representation of the world which is built into our visual system is partially independent from our general knowledge. We are more likely to see a stimulus as convex if it is a face and if it is upright (Hill and Johnston, 2007), indicating that a relatively high level of processing is at the source of the perceptual bias, yet, the same perceptual bias is not necessarily overridden by the explicit knowledge that the object is concave (e.g., Gregory, 1997). Notice however that there is evidence that being told about the possible interpretations of an ambiguous stimulus might promote its perceptual bistability (Girgus et al., 1977). Another well-known example of knowledge built into our visual system is the assumption that scenes are illuminated from above and by a single light source (e.g., Ramachandran, 1988). Objects are usually perceived as concave or convex as soon as this geometrical structure predicts a shading pattern compatible with the scene being illuminated from above (Figure 1E). The dominant percept cannot be easily overridden voluntarily, however, perceptual bistability can emerge if a single light source cannot be the determinant of the global pattern of shading (Papathomas and Gorea, 1990).

Besides favoring one interpretation of an ambiguous stimulus above another, the semantics of a visual object can have an impact on its access to awareness when it competes with other stimuli. This has been shown in the case of binocular rivalry, where meaningful stimuli tend to dominate over abstract stimuli (Yu and Blake, 1992) and coherent scenes have been found to dominate over incoherent ones (Mudrik et al., 2011). Notice however that the dominance duration of structurally impossible objects can be longer than the one of normal objects, and the dominance duration of non-words can exceed the one of words (Wolf and Hochstein, 2011): this indicates that expectancy violations might gain privileged access to awareness. Apart from their meaningfulness, stimuli might dominate in bistable displays because they are more desirable, a phenomenon which has been called “wishful seeing” (Dunning and Balcetis, 2013). Observers are more likely to report a percept to which a reward is associated both in ambiguous figures and in binocular rivalry (Balcetis and Dunning, 2006; Balcetis et al., 2012) and thirsty subjects are more likely to report transparency, which is an attribute of water (Changizi and Hall, 2001). The emotional relevance of stimuli has a more complex effect on their dominance in ambiguous displays. Emotional faces (Coren and Russell, 1992; Alpers and Gerdes, 2007) as well as emotional scenes (Alpers and Pauli, 2006) tend to dominate in binocular rivalry. However, using stimuli controlled for arousal and emotional valence (Lang et al., 2005), Sheth and Pham (2008) found that arousing stimuli tend to dominate when valence is equal. Instead, stimuli with positive valence (i.e., pleasant) tend to dominate at low arousal levels, while negative images dominate among high-arousal stimuli.

### Individual differences

Observers can show consistent inter-individual differences in the way they perceive bistable displays. Differences in one's life-long experience and genetic profile contribute to different switch rates and idiosyncratic biases favoring one percept over another in healthy subjects, and various clinical conditions can be associated with altered patterns of bistable perception. We first review some examples of studies investigating individual differences in bistable perception within the general population and then consider studies dedicated to clinical populations.

The rate at which perception oscillates between the different interpretations of a bistable stimulus is a highly stable and inheritable attribute of an observer. A study of monozygotic and dizygotic twins suggested that 52% of the between-observer variance in the rate of switching in binocular rivalry can be attributed to genetic factors (Miller et al., 2010). A similar pattern of inheritability can be observed for the switch rate in the perception of the Necker cube (Shannon et al., 2011). An individual's rate of switching when viewing bistable displays can also correlate with other stable traits. A first example of such a trait is handedness. Christman et al. (2009) compared the perception of ambiguous figures in strong right-handers and mixed-handers. Mixed-handers showed a higher rate of spontaneous reversals and switched their percept more readily as the evidence in favor of one interpretation increased. The increased strength of interhemispheric connections associated with mixed-handedness might contribute to the faster switching of bistable perception. Indeed, pathologies in the development of callosal connections have been associated with a lower reversal rate of ambiguous figures in children (Fagard et al., 2008). Some individual differences in bistable perception can be connected to other anatomical features including the structure of parietal cortex. In particular the gray matter density in the right superior parietal lobule has been found to correlate with an observer's switch rate when viewing ambiguous structure-from-motion (SFM) stimuli (Kanai et al., 2010, 2011). However, a larger share of the inter-individual differences in bistable perception might be associated with the functioning of neurotransmitter systems. It has long been known that psychoactive drugs have an effect on bistable perception (e.g., George, 1936), and there is now converging evidence for a role of dopaminergic, GABAergic and serotoninergic systems. Dopamine antagonist chlorpromazine reduces the reversal rate of ambiguous figures (Phillipson and Harris, 1984) and part of the inheritance pattern of the individual differences in reversal rate when viewing ambiguous SFM stimuli might be related to genes associated with dopaminergic transmission (Schmack et al., 2013). Van Loon et al. (2013) recently showed that higher concentrations of GABA in visual cortex are related to slower switch rates both in binocular rivalry and in ambiguous SFM stimuli, and GABA_A_ agonist Lorazepam induces a reduction in the switch rate. The rate of switching in binocular rivalry can also be modulated by the administration of serotonergic drugs (Carter et al., 2005a,b, 2007; Nagamine et al., 2008) and altered serotoninergic transmission, as well as accelerated binocular rivalry, have been reported in anxious individuals, although the relationship between the two manifestations might be quite complex (Nagamine et al., 2007, 2008).

Besides inter-individual differences in switch rate, observers can differ in their biases to perceive one particular interpretation of a bistable display (Mamassian and Wallace, 2010). Biases in bistable perception can be associated with various permanent characteristics of the observer. One example is gender. Schouten et al. (2010) investigated how male and female observers perceive ambiguous point-light walkers, structure-from-motion displays which can appear to face toward or away from the observer (Figure 1F). They found that a walker of a given gender was more likely to be perceived as facing the viewer by observers belonging to the same gender. Moreover, in general male observers were more likely to perceive a gender-neutral walker to face them as compared to female observers. The observer's gender also has more subtle consequences in the ambiguous point-light walker task, as evidenced by the fact that male observers are more sensitive to perspective cues disambiguating the facing direction (Schouten et al., 2013). Another trait which can be associated with a bias in perceiving point-light walkers is social anxiety (Van De Cruys et al., 2013). Observers with high social anxiety are more likely to report seeing the walkers pointing away from them as compared to individuals with lower social anxiety. Under those conditions, observers with high anxiety appear to be biased to perceive the less threatening interpretation of the bistable stimulus. The opposite bias however can be observed under different conditions. In particular, Gray et al. (2009) reported that anxious observers were more likely to report a face with a threatening expression as the first percept in binocular rivalry.

Multiple pathological conditions can be associated with altered perception of bistable stimuli. An abnormal pattern of bistable perception can be related to disturbances in basic aspects of the functioning of the central nervous system. In some psychiatric conditions, however, the disturbances are specifically related to the meaning or affective value of the bistable stimuli. One example of the first category is the aforementioned reduction in the reversal rate of ambiguous figures in children with agenesis of the corpus callosum (Fagard et al., 2008). Adults with autism have also been reported to exhibit altered dynamics in binocular rivalry. Specifically, autistic adults reported mixed percepts for longer times and had lower switch rates compared to healthy observers (Robertson et al., 2013). A similar pattern has been described for migraine patients (Wilkinson et al., 2008), who also exhibit less frequent perceptual switches when tested monocularly with rivalrous plaid motion stimuli (Mckendrick et al., 2011). The lower switch rate in migraine patients could be related to reduced serotonin levels in sensory cortex. Perhaps the most clearly established link between a clinical condition and bistable perception is the case of the reduced alternation rate in binocular rivalry in patients with bipolar disorder (Pettigrew and Miller, 1998; Miller et al., 2003; Vierck et al., 2013), and specifically in patients with bipolar I disorder (Nagamine et al., 2009). “Sticky” binocular rivalry is so evidently associated with bipolar disorder that it has been suggested as a possible endophenotype of the pathology (Ngo et al., 2011). The reduction in reversal rate is present albeit less prominent also when patients with bipolar disorder are tested in an ambiguous SFM (see Figure 1G) task (Krug et al., 2008).

Examples where disturbances of bistable perception emerge as a function of the cognitive attributes of the stimuli include a possible reduction in the rate of spontaneous switches in children with autism spectrum disorder while viewing ambiguous figures (Sobel et al., 2005), which has been attributed to a general deficit in executive functioning, although the evidence in this respect is not univocal (Ropar et al., 2003; Wimmer and Doherty, 2010). Disturbances related to the emotional valence of bistable stimuli have been described in generalized social anxiety disorder. Patients affected by this disorder tend to have shorter dominance duration in binocular rivalry for smiling faces as compared to healthy observers and patients with generalized anxiety disorder, consistent with a reduced sensitivity to positive social cues (Anderson et al., 2013). Patients with generalized social anxiety disorder have also been reported to have a bias to perceive fearful faces as the first percept in binocular rivalry, although they tend to maintain the fearful percept for a shorter time as compared to neutral faces (Singer et al., 2009). Generally speaking, it appears that individuals with pathological levels of social anxiety tend to preferentially perceive stimuli associated with positive social interactions. This pattern bears resemblance with the aforementioned tendency to preferentially perceive the less threatening interpretation of social stimuli in healthy individuals with high social anxiety (Van De Cruys et al., 2013).

### Learning effects

Our perception of ambiguous stimuli can be shaped by innate properties of our visual system and by our life-long experience. It is however also possible to show robust learning effects in ambiguous perception within the limited time frame of a psychophysical experiment. The choice of presenting learning effects in the section devoted to the stable characteristic of the observer is somehow arbitrary, as their lifetime can be very diverse and vary according to the characteristics of the observer and of the experimental paradigm. However, since there is experimental evidence for learning effects that last over 4 weeks, we decided to deal with their impact on ambiguous perception in this section. The question of how learning can shape perception is a long-lived one (e.g., Ammons, 1954), and evidence for learning effects in ambiguous perception emerged relatively early. A longstanding finding (Brown, 1955; Spitz and Lipman, 1962; Long et al., 1983) is that the reversal rate of a spinning ambiguous figure such as the Necker cube increases through an experimental session even when breaks take place between blocks of trials.

Besides being manifest in the evolution of reversal rate, learning effects in bistable perception can determine which percept is most likely to dominate. Chopin and Mamassian (2012) tested the dependency of the percept reported in a given trial of binocular rivalry upon the exposure to unambiguous stimuli in the preceding trials, across multiple time intervals. They observed negative correlations across short intervals and positive correlations over long intervals. They suggested that both adaptation and statistical learning take place over different time frames, both contributing to bias perceptual dominance. Ocular dominance in binocular rivalry can also show relatively enduring plasticity effects. Lunghi et al. (2013) tested observers in a binocular rivalry setting after 150 min of monocular patching. They found a relative increase in the dominance duration for the deprived eye which endured for at least 90 min.

Haijiang et al. (2006) were able to show that observers can be conditioned to use an otherwise irrelevant stimulus (e.g., stimulus position) as a cue to disambiguate an ambiguous SFM stimulus. This effect might be mediated by relatively low-level structures as the relevant position appears to be coded in a retinotopic frame of reference (Harrison and Backus, 2010) and is still lingering 4 weeks after training (Harrison and Backus, 2014). On the other side, preexposure to the ambiguous stimulus can block conditioning (Van Dam and Ernst, 2010). Conditioning of one interpretation has also been observed for static stimuli with an ambiguous three-dimensional structure (Jain and Backus, 2013).

## Transient states of the observer and ambiguous perception

### Voluntary control of bistable stimuli

As noted in the introduction, a crucial query as to the issue of cognitive influence on ambiguous perception is whether we can exert some form of control over what is currently perceived. This intriguing question appears to be inherently rooted in the topic of bistable perception: for instance, Wheatstone himself immediately after his first description of binocular rivalry noted that the observer could not determine which stimulus was perceived at will (Wheatstone, 1838). Instead, Helmholtz claimed he could exert full control over both binocular rivalry and reversible figures (Helmholtz, 1925) by paying attention to either of the alternative interpretations. The question attracted the interest of early prominent scholars: Hering (1964) and Breese (1899) maintained that perceptual dominance during rivalry was substantially determined by eye movements and a similar point of view was endorsed by Necker after his observation of the perspective reversibility of the wire cube (Necker, 1832). According to Vicholkovska (1906), Wundt also sustained that the appearance of geometric reversible figures (i.e., figures that can be reversed on the basis of reference frame re-alignment, such as the Necker cube) depended on eye movements. He even indicated the precise figural elements that needed to be fixated in order to obtain a specific form of inversion, thus supporting a “physiological” interpretation. Hering (1964) pointed out the role of low-level stimulus features (such as changes in light and shade) in producing inversions of geometric reversible figures and he also noted that practice could produce accelerations in reversal rate: he concluded that the phenomenal appearance of such figures was largely under the observer's volitional control.

Ever since these early debates, the issue of voluntary control of ambiguous stimuli has recurrently attracted the interest of scientists until today (e.g., Washburn and Gillette, 1933; Washburn et al., 1934; Pelton and Solley, 1968; Lack, 1978; Peterson and Hochberg, 1983; Struber and Stadler, 1999; Suzuki and Peterson, 2000; Toppino, 2003; Meng and Tong, 2004; van Ee et al., 2005; Chong et al., 2005; Klink et al., 2008; Hugrass and Crewther, 2012; Stonkute et al., 2012). Among such studies a distinction can be traced between the observers' ability to switch between two alternative percepts and the ability to hold either of the two in visual awareness, as changes in reversal rate can occur without variations in relative dominance of either percept (van Ee et al., 2005).

#### Control of reversal rate

There is overall agreement regarding observers' capacity to control alternation rate across a large variety of bistable stimuli: Pelton and Solley (1968) found that the instruction to switch between alternative perspectives of the Necker cube as often as possible yielded a significantly greater number of reversals than the instruction to hold a perspective (any of the two) as long as possible; Seth and Reddy (1979) also reported that observers were able to comply with different task instructions about the reversal rate of Vase-Face, Schröder Staircase and Reversible Book figures (see Box 1); Rock et al. (1994) even proposed that observers do not reverse ambiguous figures if their intention to do so is contrasted by a different intention (although different interpretations of their findings are viable). George (1936) investigated the effects of two drugs, caffeine and sodium amytal, on observers' ability to control the reversal rate of bistable figures and binocular rivalry: he found that both drugs affected rivalry less than reversible figure perception and that voluntary control of rivalry was possible, but very limited compared to figure reversal. Meredith and Meredith (1962) reported significant effects of different instructional conditions on the alternation rate of binocular rivalry, an observation that subsequently found further empirical support (Lack, 1978; Meng and Tong, 2004; van Ee et al., 2005); Kohler et al. (2008) investigated apparent motion perception (Figure 1H) and described a halving in percept duration under the requirement to switch as quickly as possible between alternatives as opposed to passive viewing conditions.

Box 1 **Glossary.**

**Table:** 

**Phenomenon**	**Figures**	**Description**	**References**
Hollow-face illusion	1A	A concave mask is perceived as a convex face.	Gregory, 1997
Reversible figures	1B,C	The stimulus configuration allows for multiple interpretations. Within the category of reversible figures, a difference can be traced between figures whose reversibility is based on reference frame re-alignment, such as the Necker cube (**1B**, other instances are the Schröder Staircase and the Reversible Book) and on meaning reconstruction, such as the Wife/Mother-in-law ambiguous figure (**1C**, other instances are Rubin's vase/face and the duck/rabbit figure).	Long and Toppino, 2004
Binocular rivalry	1D	It occurs when two different images are shown simultaneously to the two eyes at a corresponding retinal location. Perception switches between the monocular inputs, although phenomena of mixed dominance are also possible (piecemeal rivalry).	Wheatstone, 1838; Levelt, 1968
Shape from shading	1E	Objects are perceived as concave or convex depending on whether the shading pattern is compatible with the scene being illuminated from above.	Ramachandran, 1988
Ambiguous point-light walker	1F	A human walker can be defined only by dots placed at the main joints: the dots configuration is immediately perceived as a walker as soon as it starts moving (i.e., it is an instance of structure-form-motion, see **1G**). Bistable point-light walkers are compatible with two three-dimensional interpretations, dissimilar only for the depth order of the body parts: a point-light walker in frontal view can appear to face toward or away from the observer.	Vanrie et al., 2004; Manera et al., 2012
Ambiguous structure-from-motion	1G	The stimulus consists of a two-dimensional projection of a three-dimensional object (in this case a sphere), composed of dots laying on its imaginary surface: when motion information is added to the display, the stimulus is seen as a three-dimensional object and opposite directions of motion alternate in perception.	Metzger, 1935
Ambiguous apparent motion	1H,I	The repetitive presentation of single dots at a fixed frame rate induces the impression of motion: rather than perceiving static dots flashing at different positions on the screen, the viewer perceives them moving. The intermittent and subsequent presentation of four dots induces the subjective impression of vertical or horizontal motion in Figure 1H, whereas in Figure 1I the two dots may appear to translate horizontally or to expand and contract (loom and recede)	Wertheimer, 1912; Suzuki and Peterson, 2000; Kohler et al., 2008
Plaid motion	1J	When two drifting sinusoidal gratings are superimposed, the viewer may perceive the motion of the single semi-transparent gratings. Alternatively, what is perceived is a rigid structure (a plaid) drifting in a direction determined by the velocity and direction of the components.	Adelson and Movshon, 1982

However, voluntary control of reversal rate seems to be limited both by physiological and stimulus constraints. On one hand, it does not seem to be in the power of will to fully prevent alternations (but see Carter et al., 2005b for the effects of intensive and prolonged meditation training on binocular rivalry in Buddhist monks) and it is often hardly possible to precisely choose the moment of reversal: as a matter of fact, phenomenal reversals are physiologically bound to neural mechanisms of adaptation and mutual inhibition between neural populations representing alternative interpretations (e.g., Blake, 1989; Van Ee, 2009; Shpiro et al., 2009; Seely and Chow, 2011). On the other hand, control of alternation rates is related to the level of processing allowed by the physical characteristics of the stimulus. For instance, within the category of reversible figures, Struber and Stadler (1999) noted that observers exerted greater control of alternation rates for those stimuli whose ambiguity could be solved by reconstruction of meaning rather than by reference frame re-alignment (e.g., the duck/rabbit figure as opposed to the Necker cube, see Box 1). In binocular rivalry, van Ee et al. (2005) reported greater ability to control higher level stimuli such as houses and faces than sinewave gratings, and a recent study of Hugrass and Crewther (2012) showed that whereas the reversal of stationary gratings is resistant to voluntary control, the introduction of motion information (both in the form of apparent and of real drifting of the gratings) seems to enable the observers to generate voluntary alternations. The authors maintained that this effect cannot be attributed to the mere control of eye movements (at least of saccadic ones) during the presentation of motion stimuli, because their frequency decreased when the experimental task involved voluntary control of switches and because their rate did not change at the time of perceptual reversals. However, one may speculate that smooth pursuit movements could aid observers in controlling perceptual alternations. Furthermore, Alais et al. (2010) found that diverting attention to a secondary task slowed perceptual alternations differently for different types of bistable stimuli, with the Necker cube and house/face rivalry being more amenable to attentional modulation than rivalry gratings.

#### Control of relative dominance

Certainly, the issue of mastery over ambiguous perception has been explored not only in terms of reversal rate control, but also in terms of the ability to maintain a specific percept in visual awareness. This issue has been largely investigated by explicitly requiring participants to “hold” one of the two alternative interpretations: this is one typical instruction employed to test the effects of focused endogenous attention, but other instructional sets and experimental paradigms have been employed more recently and will be reviewed in the next section.

There is wide agreement on the observers' capability to augment the proportion of time they perceive one of two alternatives, as instructed, in the field of ambiguous figures. Experimental reports of the effectiveness of the “hold” instruction have been described for the Necker cube (Washburn and Gillette, 1933; Washburn et al., 1934; Mull et al., 1952; Peterson and Hochberg, 1983; Gomez et al., 1995; Toppino, 2003; Kornmeier et al., 2009) and for other perspective reversible figures (Washburn et al., 1934; Liebert and Burk, 1985). Further efforts were made to demonstrate that the effect of the instructional set is genuinely perceptual rather than ascribable to response biases by employing reversing stereograms (Peterson, 1986) and three-dimensional Necker cubes (Hochberg and Peterson, 1987) as stimuli and recording indirect measures that were perceptually coupled to the one of interest. In the case of reversing stereograms, the viewer observed through a stereoscope a stereo pair of images containing a central square area that could appear in front of or behind the surrounding area. The authors asked participants to hold one depth organization and to respond about perceived depth directly or indirectly via a judgment about (illusory) parallactic motion of the central relative to the surrounding area. Since depth ordering and motion parallax are perceptually coupled, the systematic covariation between the two kinds of responses was interpreted against a post-percetual explanation of instructional set effects. Likewise, in a three-dimensional rotating Necker cube the direction of motion (clockwise or counterclockwise) is perceptually coupled with depth ordering: the relative shifting of the front and rear faces of the cube defines clockwise or counterclockwise rotation (Hochberg and Peterson, 1987). The authors asked participants to report the cube direction of rotation as an indirect measure of perceived depth organization. The studies of Peterson and colleagues also helped clarifying the relationship between voluntary control of reversible perspective and fixation of specific figural elements: although fixation instructions influence subjective reports, they cannot fully account for voluntary control of perceptual dominance (Peterson and Hochberg, 1983; Hochberg and Peterson, 1987), and neither can vergence eye movements (Peterson, 1986). Furthermore, Peterson and Gibson (1991) found that focusing spatial attention to subregions of the Necker cube can influence its perceived depth organization regardless of fixation location when observers are required to hold one interpretation. However, Toppino (2003) argued that the effects of intention cannot be merely ascribed to the selection of appropriate focal features within the stimulus for primary processing. Other kinds of ambiguous stimuli have proven amenable to voluntary control: Hol et al. (2003) examined ambiguous structure-from-motion and noted that the orthographic projection of a rotating transparent cylinder leads to more perceptual alternatives than have been classically studied. Besides a three-dimensional cylinder revolving clockwise or counterclockwise, observers were able to perceive two convex half-cylinders (and, to a lesser extent, two concave ones) one in front of the other. Importantly, the amount of time during which a certain percept was present could be significantly biased by voluntarily attending to it. Suzuki and Peterson (2000) investigated ambiguous apparent motion stimuli that could be interpreted either as translational or as expansion/contraction movements (Figure 1I) and observed that voluntary control increased the probability to see the instructed motion in a multiplicative way. The authors manipulated stimulus eccentricity and orientation so as to favor one interpretation under passive viewing conditions: they found that the effect of intention was greater the stronger the configuration bias. Similar dependencies between physical stimulus parameters and strength of voluntary control were reported by Brouwer and van Ee (2006) and by Klink et al. (2008) for ambiguous structure-from-motion and binocular rivalry, so that Klink et al. (2008) proposed a model to account for an early locus of interaction between top-down control and sensory processing, which would occur prior to the resolution of perceptual conflict.

Voluntary control of dominance under “hold” instructions has been reported also during binocular rivalry (Washburn and Gillette, 1933; Collyer and Bevan, 1970; Lack, 1978; Meng and Tong, 2004) and a series of studies conducted by Lack (1978) ruled out that the effects of intention could be explained in terms of mere control of accommodation, eye movements, eye blinks, or pupil constriction. However, the ability to voluntarily control rivalry seems to be somehow limited, especially if compared to other instances of bistable stimuli (Washburn and Gillette, 1933; George, 1936; Meng and Tong, 2004). Meng and Tong (2004) compared observers' performance during the display of binocular rivalry and Necker cube stimuli: they found that observers could effectively modulate reversal rate for both rivalry and the Necker cube, but their control of rivalry dominance was very weak, at variance with performance for the Necker cube. The authors concluded that selective attentional control of rivalry is rather poor and that mastery over reversal rate relies on strategies other than selective attention.

### Voluntary attention

This section focuses on the effects of voluntary attention deployment on the perception of ambiguous displays, whereas the effects of involuntary or exogenous attention will not be covered and have been reviewed elsewhere (e.g., see Dieter and Tadin, 2011; Paffen and Alais, 2011 for a discussion of the topic in the domain of binocular rivalry).

Whether the deployment of attention can modify the appearance of ambiguous stimuli is another topic that has attracted the interest of scientists both in the past and in more recent times. Maybe the first question to be addressed was whether a percept could gain preferential access to consciousness if the observer focused his/her attention on it (Helmholtz, 1925). Indeed, the issues of voluntary control, intention, and endogenous selective attention are overlapping to a great extent (e.g., Suzuki and Peterson, 2000; Meng and Tong, 2004) and have been typically investigated by instructing observers to “hold” one of the alternative percepts, as reviewed above. However, endogenous selective attention can be manipulated in different ways. For instance, Ooi and He (1999) indicated to observers which stimulus to attend to by means of a visual cue that was continuously visible and occupied a different spatial location than the rival target: this prevented suppression of the dominant eye when a transient stimulus was displayed to the non-dominant eye, which otherwise typically occurs (Grindley and Townsend, 1965; Duensing and Miller, 1979; Ooi and He, 1999). More recent studies (Chong et al., 2005; Chong and Blake, 2006; Hancock and Andrews, 2007) demonstrated that requiring participants to track changes in one of the rival stimuli impacts both initial selection (Chong and Blake, 2006) and rivalry dominance (Chong et al., 2005; Hancock and Andrews, 2007), and does so in a way that can be likened to increases in the cued stimulus contrast (Chong et al., 2005; Chong and Blake, 2006). Interestingly, these findings match Helmholtz's observation that he could hold a rival stimulus dominant by counting the lines present in its display.

Another way the allocation of attention may affect ambiguous perception is by diverting cognitive resources to a concurrent task. This issue was first studied in the domain of ambiguous figures: Reisberg (1983) reported increased latencies of first reversals when observers were engaged in a distractor task and Reisberg and O'Shaughnessy (1984) further showed that withdrawing attention from the bistable stimuli slows reversal rates, thus indicating that cognitive resources are implicated not only in the discovery of the alternative construal, but also in the processes causing switches between the alternative interpretations. In another experiment of their influential study about the effects of attention on binocular rivalry, Ooi and He (1999) displayed a rectangular frame to one eye in order to direct attention on the framed grating of the rivalry pair and asked participants to report the color of the perceived grating: this kind of cuing was shown to increase dominance of the cued eye in a previous experiment of their study. They further required participants to perform a concurrent Vernier task at a different spatial location, and observed that the dominance induced by the cuing procedure was strongly reduced under divided attention conditions. More recently, Paffen et al. (2006) found that diverting attention from binocular rivalry to a concurrent visual task reduced alternations in a way that was proportional to task difficulty and that mimicked the lowering of the rival stimuli contrast: they proposed that the effect of attention on rivalry dynamics is indirect and achieved through the enhancement of the effective contrast. Similar conclusions were drawn by Paffen and Hooge (2011) in the context of distributed attention to multiple rivalry-inducing elements and, as noted above, by Chong et al. (Chong et al., 2005; Chong and Blake, 2006) about the effects of endogenous selective attention as elicited by a tracking task. Interestingly, Alais et al. (2010) tested whether a slowing of reversal rate occurs when attention is diverted to a concurrent auditory task and observed that the number of reversals decreased as a function of task difficulty, albeit at a different rate for different kinds of bistable stimuli. This finding favors an interpretation of the effect of diverted attention in terms of a withdrawal of supramodal cognitive resources from the perceptual task, rather than a sensory-specific phenomenon. This is consistent with the aforementioned work of Reisberg and colleagues on reversible figures (Reisberg, 1983; Reisberg and O'Shaughnessy, 1984), which showed that alternation rates slow and latencies of the first reversal increase when participants are engaged in cognitive demanding task such as counting backwards or memorizing digits for subsequent recall.

Analogous slowing of alternation rates has been reported for reversible stereograms (Peterson, 1986), apparent motion (Kohler et al., 2008), ambiguous structure-from-motion (Pastukhov and Braun, 2007) and drifting plaids (Figure 1J, Pastukhov and Braun, 2007) when attention is deployed to a concomitant task at fixation. Pastukhov and Braun (2007) employed intermittent presentation of stimuli, and found that reversals became less frequent but still occurred when attention was prevented from shifting to the ambiguous stimulus: the authors thus excluded that attention is necessary to trigger phenomenal reversal. A negligible role of attention in triggering phenomenal reversals during intermittent presentation of Necker lattices (i.e., grids made of juxtaposed Necker cubes) has been evidenced also by Intaitè et al. (2013). Instead, opposite conclusions have been drawn by Brascamp and Blake (2012) in the field of binocular rivalry. The authors employed a flash suppression paradigm to force visibility of one of the rival stimuli at the beginning of each trial and then compared observers' reports on rivalry after a delay during which: (a) participants performed an attentionally demanding task at fixation while the rivalry stimulus was still present on screen, (b) participants performed the same demanding task in the presence of non-rival stimulation, (c) participants monitored rivalry disregarding the stimuli at fixation. They found a spillover of the flash-suppression procedure on subsequent rivalry dominance in (c) but not in (a) nor in (b): observers' performance was comparable when the rivalry stimulus was physically removed and when it was unattended, thus suggesting that full attention withdrawal abolishes rivalry. The discrepancy between the results of Pastukhov and Braun (2007) and Intaitè et al. (2013) and the results of Brascamp and Blake (2012) could be due on one hand to a difference between the types of stimuli inducing the perceptual conflict; on the other hand, the experimental manipulation disrupted visual awareness of the ambiguous stimuli in Brascamp and Blake's, but not in Pastukhov and Braun's nor in Intaitè and colleagues's study. Interestingly, Stonkute et al. (2012) showed that the withdrawal of spatial attention can also produce qualitative changes in the interpretation of a bistable stimulus. The authors studied the impact of sudden inversions of planar flow motion on the perception of ambiguous structure-from-motion spheres: when all the dots composing the sphere reverse their motion direction, the perceptual outcome could be either an inversion of the illusory depth of each dot, with motion direction being maintained, or an inversion of motion direction, with conservation of illusory depth. When attention is diverted to a demanding task at a different spatial location, reversals of illusory motion drop in favor of reversals of illusory rotation.

### Visual working memory and imagery

Experimental manipulations of voluntary attention and intention have been by far the most studied ways to test how the state of the observer can be transiently changed and how this change affects ambiguous perception. One other remarkable and ever-changing characteristic of the observer's mental state are the contents of working memory (WM). Visual WM is a crucial factor subtending visually guided behavior, and the effects of retaining a visual stimulus in WM on the processing speed of images displayed during the retention interval has been widely investigated (e.g., Downing, 2000; Robinson et al., 2008; Turatto et al., 2008; Pan and Soto, 2010). More recently, the contents of working memory have been shown to have a direct impact on how visual stimuli appear to the observer: the storage of low-level visual features in WM can produce systematic misperceptions of currently presented stimuli (Kang et al., 2011; Scocchia et al., 2013a). The effect of visual WM contents on bistable perception, however, is not a conclusive one. Rather, its impact seems to depend on the kind of stimuli being investigated: Scocchia et al. (2013a) engaged participants in a delayed discrimination task, asking them to memorize the speed of an unambiguous structure-from-motion sphere, and displayed an ambiguous sphere during the retention interval. The perceived direction of motion of the ambiguously rotating sphere was biased toward a match with the memorized one. The situation seems to be different for binocular rivalry, however, since holding a visual item in WM was not sufficient to bias rivalry (Scocchia et al., 2014). As one of the experiments described by Scocchia et al. (2014) employed an experimental paradigm very similar to the one of the structure-from-motion study (Scocchia et al., 2013b), the divergent results are unlikely due to methodological discrepancies. Rather, they could be ascribed to different neural mechanisms involved in different kinds of ambiguous perception, with binocular rivalry being based on competition at earlier stages of visual processing. Indeed, explanations of binocular rivalry in terms of early-level competition have met both empirical and theoretical consent (e.g., Blake, 1989; Haynes et al., 2005; Lee et al., 2005; Seely and Chow, 2011) and, consistently with the outcome of Scocchia and colleagues, previous studies comparing binocular rivalry to other instances of bistable perception showed less amenability of rivalry to top-down modulation (i.e., Washburn and Gillette, 1933; George, 1936; Meng and Tong, 2004).

The question as to whether memory and imagery can influence ambiguous perception was posed by Horliz and O'Leary (1993) in the domain of figure-ground reversible figures. The authors reported the effects of training participants to imagine the alternative interpretation of a figure displayed with disambiguating cues on subsequent perception of reversible figures. They observed that the results yielded by this training procedure were comparable to those of a condition where participants were exposed prior to testing to the different (disambiguated) alternatives of the same reversible figures employed during testing. Both conditions differed in reversal rate and first reported percept from a control condition where participants received training with ambiguous figures that were not employed during testing. The authors discussed the data in terms of high-level contributions from imagery and memory to bistable perception. However, given that imagery training was conducted on figure-ground reversible stimuli other than the ones employed during testing and that overall dominance was not affected by the experimental manipulations, such contributions likely operated indirectly, with participants learning how to switch between alternative interpretations. Indeed, control of alternation rates can be improved by training (e.g., Lack, 1978). More convincing evidence for a role played by imagery in bistable perception has been obtained by Pearson et al. (2008) in the domain of binocular rivalry. The authors employed intermittent presentation of rivalry grating stimuli, which is known to reduce the number of alternations thus leading to perceptual stabilization (Leopold et al., 2002; Pearson and Brascamp, 2008). Participants had to perform an imagery task during the blank interval between subsequent rivalry presentations: they were required to visualize in the eye of their mind either the stimulus that was dominant or the one that was suppressed during the previous presentation.

This manipulation led to a disruption of perceptual stability when the imagined grating matched the non-dominant one. The effect was comparable to the one of faint physical stimulation displayed during the blank interval and increased as a function of the temporal duration of the imagined or faint physical stimulus, in a very similar fashion. Furthermore, the authors observed that the impact of imagery on binocular rivalry is location and orientation specific. These results may seem at odds with the ones of Scocchia et al. (2014), who failed to observe visual WM effects on rivalry: if on one hand such a discrepancy may be explained in terms of the different perceptual dynamics underlying prolonged and continuous vs. brief and intermittent rivalry presentation (Dieter and Tadin, 2011), on the other hand the effects of visual WM and imagery on binocular rivalry need not be the same. As a matter of fact, mental images can be evoked in the absence of any sensory input (e.g., Kosslyn, 1973; Kosslyn et al., 2000), whereas visual WM requires active encoding and maintenance of physical items in order to prevent memory decay (Baddeley, 2003). Furthermore, there is evidence that dynamic visual noise (e.g., arrays of dots flickering at a predetermined rate and occupying random positions in the visual space) disrupts performance during imagery but not visual WM tasks (Andrade et al., 2002; Baddeley, 2007), and that TMS applied to V1/V2 brain areas can selectively impair performance in visual WM but not in imagery tasks (Cattaneo et al., 2009): this suggests that visual WM and imagery may rely on different operating mechanisms that relate to perception in different ways.

### Prior history of perceptual dominance

Another important and thoroughly studied aspect of how transient states may influence ambiguous perception is recent stimulation and dominance history. Probably the most straightforward effect of recent stimulation on the dynamics of ambiguous perception is the one of adaptation (i.e., repulsion toward the opposite percept). However, if the repulsive effects of prolonged monocular adaptation (Blake and Overton, 1979; Blake et al., 1990; van Boxtel et al., 2008) and of extended viewing of unambiguous displays (Wolfe, 1984; Nawrot and Blake, 1989; Petersik, 2002) on subsequent ambiguous perception have clearly been established, the adaptation effects of subjective dominance periods within conventional ambiguous displays have long been elusive. In this case, one would expect dominance durations to diminish after extended periods of dominance of the same percept: yet, several studies that examined interdependence between successive dominance epochs failed to observe significant correlations (e.g., Fox and Herrmann, 1967; Lehky, 1995; Logothetis et al., 1996). Only recently Pastukhov and Braun (2011) noted that the *cumulative history* of past perceptual experience can predict future dominance within the time frame of an experiment with ambiguous displays: dominance durations of both rivalry and ambiguous structure-from-motion show a robust correlation with the integral of past perceptual states, weighted toward the most recent states. In the domain of binocular rivalry, Kang and Blake (2010) further showed that physically removing the suppressed image and reintroducing it after a brief interval increases its subsequent dominance durations: dominance durations decrease monotonically as a function of adaptation durations.

The dependency of current ambiguous perception from prior history of perceptual dominance becomes evident when one considers the effects of interleaving brief presentations of bistable stimuli with blank periods of several seconds (Leopold et al., 2002; Pearson and Brascamp, 2008): in these conditions, reversal rates have been reported to slow by two orders of magnitude compared to continuous viewing, meaning that a sensory trace of the image is temporarily stored across presentations and helps stabilizing the subjective appearance of a wide range of ambiguous stimuli. Interestingly, this form of priming does not run out after each single stimulus presentation, but builds up over time (Maloney et al., 2005; Brascamp et al., 2008; Pastukhov and Braun, 2008). For instance, Brascamp et al. (2008) interleaved intermittent and continuous presentation of ambiguous stimuli with the aim to disrupt stabilization during continuous viewing by means of flash suppression: they found that, when intermittent presentation resumed, current dominance could be predicted on the basis of the dominance ratio between percepts during the previous minute of stimulus presentation. Likewise, Pastukhov and Braun (2008) showed that perceptual stabilization of structure-from-motion displays depends on an history of three or more dominance periods. A further study of Brascamp et al. (2009) examined intermittent presentation of ambiguous stimuli on a longer timescale than the ones usually reported in empirical research: they observed that intermittent presentation, rather than definitively stabilizing perception, produces reversals at fairly regular intervals which seem to depend on the duration of the blank period: the authors propose that intermittent presentation causes a perceptual reversals cycle on a much longer time scale compared to the alternations observed during continuous presentation.

Finally, it should be noted that the effects of perceptual priming and adaptation might be underpinned by different neural representations, as proposed by Pastukhov and colleagues to account for their results (Pastukhov et al., 2013a, 2014). The authors employed the same intermittent structure-from-motion display to test the effects of perceptual priming and adaptation on the subjective appearance of the bistable stimulus: whereas priming depended on the three-dimensional shape of the object rather than on its volume, the opposite pattern of results was observed for perceptual adaptation, which depended on the volume rather than on the shape of the object. The authors suggested that the neural populations responsible for adaptation may be localized relatively high in the visual processing hierarchy (i.e., area MST), so that they may code for illusory rotation in depth without preserving information about shape.

## Modeling ambiguous perception: toward accounting for the impact of stable and transient states of the observer

In principle, computational models can be formulated at very different levels of abstraction and with different goals in mind. At one extreme end, simple *phenomenological models* may attempt to describe certain aspects of the experimental data in a compact and mathematically simple and elegant form. More detailed *mechanistic models* may try to establish a closer relationship to the underlying neurophysiological mechanisms and may, e.g., utilize complex networks of spiking neurons endowed with sophisticated biophysical mechanisms. Finally, there are so-called *normative models*, which try to relate the phenomenon in question to certain optimality principles. In the context of ambiguous perception, such models may ask to what extent, e.g., the perceptual switching may reflect an underlying optimal information processing strategy. Importantly, there is no clear-cut distinction between these different classes of models and any given model may carry aspects of all three classes. What is common among all models is that they try to describe and explain certain observed phenomena. In addition, they should generate testable predictions that can be used to falsify them.

### A generic model

We start our discussion by outlining a generic neuro-computational model for ambiguous perception. Many concrete instances and various extensions of this generic model have been proposed over the years, which we will not review in detail (Lehky, 1988; Wilson et al., 2001; Laing and Chow, 2002; Wilson, 2003; Seely and Chow, 2011). Instead, we will focus on a few recent extensions of the generic model that have been introduced to account for influences of stable and transient states of the observer on ambiguous perception reviewed above. Finally, we highlight some open questions and identify promising directions for future research.

**Figure 3:**
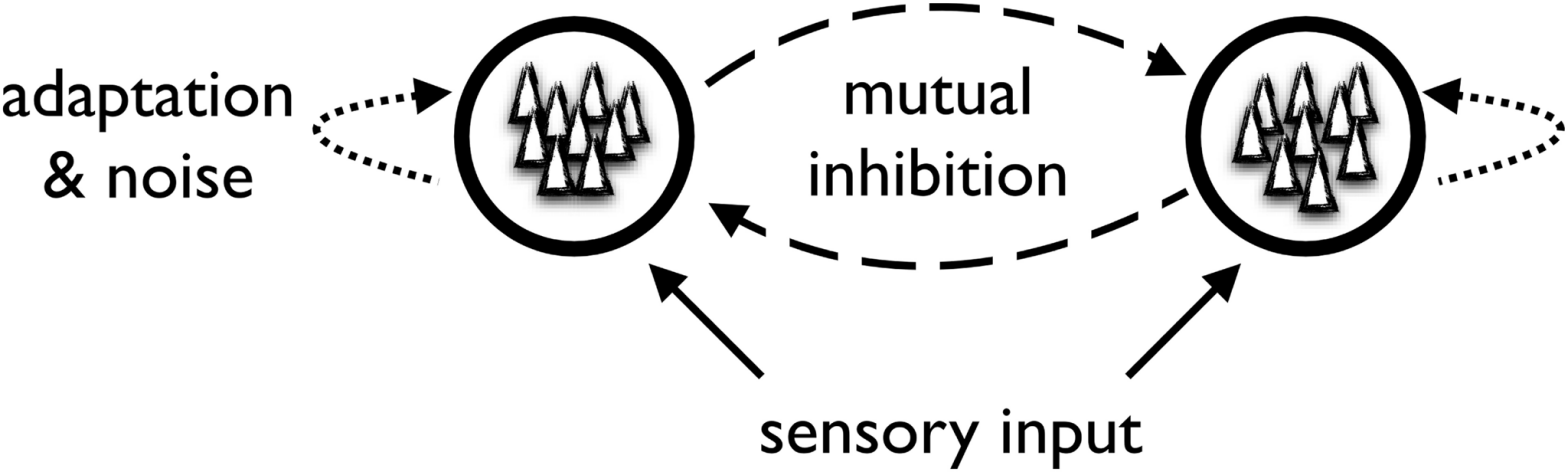
**Sketch of the generic neuro-computational model**.

At the heart of the generic model are two or more competing neural populations whose activity is linked to the perception of the different stimulus interpretations (Figure 3). For example, a model of binocular rivalry may comprise two populations of neurons corresponding to the perception of the stimuli presented to the left and right eye. Importantly, the neural populations are competing with one another in a “winner-takes-all” fashion. Only one population can be highly active at a time and while it is active, it suppresses activity in the other population. To achieve this feature, the generic model contains inhibitory couplings between the neural populations representing the different perceptual alternatives and utilizes non-linear properties of the neural populations. This results in a multistable dynamical system where one population wins the competition and thereby signals the subject's perceptual interpretation of the stimulus.

To account for the spontaneous switching between different perceptual alternatives, the generic model makes use of some form of adaptation and noise. Adaptation can be thought of as a slow mechanism of neuronal fatigue (time scale of seconds) that leads to the winning population getting “tired” and reducing its activity such that the other population can eventually take over. At the neurophysiological level, this fatigue is thought to be due to slow after-hyperpolarizing currents, but short-term depression of excitatory synaptic connections within the winning neural population may also contribute to its demise.

Deterministic mechanisms for neuronal adaptation and synaptic depression alone tend to produce regular oscillations where one population suppresses the other for a fixed amount of time before the other population takes over. Since such periodic behavior is not seen experimentally, random noise is added to the model to account for the stochasticity of perceptual switches. This allows to describe the statistical distribution of dominance times, which have been reported to approximately follow a Gamma or lognormal distribution (e.g., Levelt, 1968; Lehky, 1988). The neurophysiological basis of the noise is often assumed to be intrinsic neural noise but also eye movements can be construed as adding “noise.” Interestingly, Van Ee (2009) has shown that adding noise to the adaptation dynamics of the neural population can account for certain serial correlation data in dominance durations. Many published models are consistent with or extend this admittedly rather vague description of a generic model and they have accounted for a wide range of observed phenomena in ambiguous perception (Lehky, 1988; Wilson et al., 2001; Laing and Chow, 2002; Wilson, 2003; Seely and Chow, 2011). The generic model is therefore a good starting point for considering influences of stable and transient states of the observer on ambiguous perception.

### The roles of noise, adaptation, and inhibition in driving perceptual switches

The relative importance of noise vs. adaptation for perceptual switching has been discussed by several groups (e.g., Moreno-Bote et al., 2007; Shpiro et al., 2009; Pastukhov et al., 2013b). By performing a careful parameter analysis of various instances of the generic model, Shpiro et al. (2009) and Pastukhov et al. (2013b) have argued that the perceputal system is operating in an intermediate regime where it is neither adaptation nor noise-dominated. Specifically, Pastukhov et al. (2013b) observed that model parameters fit to data from human subjects fell in a small region of the parameter space largely overlapping with a theoretically determined “sweet spot,” where the model balances perceptual stability with sensitivity to input modulations. Their work represents an interesting step toward a normative account of ambiguous perception.

An interesting variant of ambiguous perception uses stimuli with more than just two possible interpretations. Using such stimuli allows to go beyond the question of when a switch occurs to also asking what the next percept will be, because now there is more than one alternative to the currently perceived interpretation. Huguet et al. (2014) have recently presented a model for tristable perception induced by plaid stimuli (Figure 1J). These can be perceived as either a coherently moving plaid pattern or as two semi-transparent gratings moving on top of each other in different directions. Since either grating may be perceived as lying on top of the other, there is a total of three different percepts possible. Importantly, since perception can switch from one interpretation to two distinct alternatives, the probability of switching to one or the other can provide a separate window into the mechanisms underlying ambiguous perception. They propose a model similar to the generic model outlined above but with three competing neural populations corresponding to the three perceptual interpretations: the model parameters are tuned to fit data on dominance period durations and switch probabilities. The authors conclude that the data are best accounted for by a model where random fluctuations essentially determine the dominance periods, but adaptation influences what percept will dominate next. This somewhat contradicts some of the works mentioned above which posit an important albeit not dominating role of adaptation for the time course of percept switching.

Inhibition can also affect the timing of perceptual alternations. By simulating a version of the generic model, Van Loon et al. (2013) derived the prediction that increased inhibitory interactions between the competing neural populations should lead to slower perceptual switching. They then went on to test this prediction experimentally. When estimating levels of GABA, the major inhibitory neurotransmitter in the brain, in visual areas through magnetic resonance spectroscopy, they found that GABA levels were significantly negatively correlated with perceptual alternation rates as predicted by the model. Moreover, administration of lorazepam which stimulates GABA_A_ receptors was also found to slow perceptual alternations.

The role of inhibition was also recently investigated by Hoshino (2013), who proposed a model to account for age-related differences in perceptual switching. It has been known for a long time that perceptual switching is slower in older adults compared to younger adults. Hoshino's model suggests that this difference might be caused by age-related differences in the regulation of GABA. Such modeling of the neural underpinnings of individual differences in ambiguous perception or the role of other stable states of the observer as discussed in Section Stable States of the Observer and Ambiguous Perception are promising areas for future research.

### Modeling the effects of dominance history and voluntary control

A few attempts to model transient states of the observer have been made for the effects of prior history of perceptual dominance and for those of voluntary control. As to the former, the empirical observation that interleaving briefly presented ambiguous displays with blank intervals of several seconds substantially slowed reversal rates, showed that successive dominance periods are not independent of one another as was previously believed (see Section Prior History of Perceptual Dominance). In the context of intermittently presented ambiguous stimuli, stimulus ON/OFF timing controls the generation of repeating, alternating or other more complex choice sequences. Noest et al. (2007) have proposed a minimal model that captures a wide range of these effects. Compared to the generic model outlined above, it introduces an additional facilitatory mechanism. This facilitation builds up with time to favor perceptual decisions upon stimulus onset which are congruent with the recently perceived interpretation. A similar model by Wilson (2007) uses a synaptic facilitation mechanism for the same purpose. Brascamp et al. (2008) have extended the model of Noest et al. (2007) by adding multiple time scales of adaptation. This allows them to account for findings in experiments with intermittently presented ambiguous stimuli demonstrating effects of prior dominance history extending over minutes. A similar effect is obtained by an accumulating bias in a simple descriptive model of Pastukhov and Braun (2008). Their model does not explicitly represent the time varying activity of competing neural populations as the generic model does, but is restricted to a simple description of how a *bias* for selecting one percept over another is accumulating and decaying during perception of either stimulus interpretation.

A model to account for the effect of voluntary control on ambiguous perception has been put forward by Klink et al. (2008). They introduce a two-stage model with two adapting neural populations at each of the two stages and cross-inihibition between the two populations of the higher stage. Voluntary control is modeled as a simple multiplicative gain modulation of the excitatory connections from the lower to the higher stage. Their model is consistent with the idea that top-down attentional control over bistable stimuli may work by modulating the gain of feature representations in early processing stages.

Overall, the generic model and its recent extensions have been quite successful in describing fundamental aspects of ambiguous perception and offering plausible accounts for the underlying neural mechanisms, covering many of the phenomenological and mechanistic aspects of the problem. So far, however, only few of the various stable and transients influences on ambiguous perception have been modeled. We identify this as a promising area for future research.

### Toward normative accounts of ambiguous perception

Another opportunity results since only little work has attempted to develop normative accounts of ambiguous perception phenomena. An adequate framework for this might be to treat ambiguous perception as just a special case of a generic process of statistical inference. Early on, Dayan (1998) already pointed out that the stochastic nature of switches in binocular rivalry might be related to some form of sampling-like statistical inference procedure. As sampling theories are gaining momentum in Neuroscience (Hoyer and Hyvärinen, 2003; Lee and Mumford, 2003; Shi and Griffiths, 2009; Fiser et al., 2010), it will be interesting to see if the diverse phenomenology of ambiguous perception can be seen as a reflection of an underlying approximately optimal statistical learning and inference mechanism. In this context, Hohwy et al. (2008) review binocular rivalry from the perspective of predictive coding, which assumes that lower level processing stages only signal differences between bottom-up inputs and top-down predictions to the next higher processing stage. Some concrete models that use sampling-based inference ideas to model ambiguous perception have been presented by Sundareswara and Schrater (2008), Buesing et al. (2011), and Gershman et al. (2012). Future modeling work should aim to develop a synthesized view of ambiguous perception that reconciles such normative approaches with detailed mechanistic and simpler phenomenological descriptions. It will then be interesting to see how the various stable and transient influences on ambiguous perception can be accounted for in the context of such models.

## Concluding remarks

The truthfulness of the outside world, as it appears to the viewer's eye, is hardly ever questioned by the man in the street. Despite the apparent coincidence between the phenomenal and the physical world, it is now evident that the world as we perceive it is the result of a complex process of inference operated by the brain. Whenever we face noisy, feeble or ambiguous visual stimulation, the outcome of this process is determined by the state of our perceptual and cognitive system, which, in turn, is the result of both the evolution of our species, the learning that took place during our lifespan, the recent stimulation we received and our current goals. The perception of ambiguous stimuli is thus an invaluable tool for the scientist aiming to explore the determinants of perception. In this contribution we tried to provide a comprehensive overview of the studies that investigated how the state of the observer can affect the perception of ambiguous stimuli. We reviewed both classical studies and recent contributions, and addressed different types of bistable perception, from reversible figures to binocular rivalry (examples are reported in Figure 1 and Box 1). We described a large spectrum of phenomena set to multiple time-scales, spanning from the effects of gender, handedness and genetic background, that characterize individual performance through a lifetime and beyond, to the effects of adaptation and attention, whose duration is in the range of seconds (Figure 2). Furthermore, we covered both aspects that act on perception at a physiological level, such as individual differences associated with the functioning of the neurotransmitter systems, and aspects that act at much higher level of processing, such as the contents of working memory and imagery. Finally, we reviewed current models of ambiguous perception that may conciliate the impact of the state of the observer. The available theoretical models mostly account for the low-level aspects of ambiguous perception and have focused on binocular rivalry as a paradigmatic example. Whether these models, and more generally the framework of the generic model outlined above (Figure 3), apply to the different kinds of ambiguous perception, including reversible figures, is an intriguing and yet unanswered question. Ultimately, the range of phenomena in ambiguous perception is wide enough to confront the idea that they could be accounted for within a unique framework: the challenge to face is integrating different gradients of top-down modulation in a simple, physiologically plausible framework. In this respect, assessing the impact of stable and transient states of the observer is crucial to the understanding of ambiguous perception as a whole: amenability to top-down modulation of different kinds of bistable stimuli allows to identify the level of processing at which competition between different representations occurs and helps pinpointing the mechanisms underlying such competition. In principle, different levels of top-down modulation, from stable individual differences and clinical conditions, to the aspects related to meaning and semantics, to the impact of more volatile cognitive factors, such as attentional deployment, may be modeled as acting at different levels of perceptual processing: a comprehensive explanation of ambiguous perception should integrate the important share played by the state of the observer.

The impact of the observer on what is perceived is indeed a very complex subject matter, which has been addressed from different viewpoints: up to now, the various aspects whose effect on ambiguous perception we reviewed have typically been studied in isolation. However, it is very plausible that at least some of these aspects exert a convergent influence on ambiguous perception: for instance, statistical learning of ambiguous displays likely relies on some form of visual memory, whereas visual imagery may be employed to exert voluntary control on what is currently perceived. Clarifying how these aspects interact would improve our understanding not only of ambiguous phenomena, but of the principles of perception overall. In our opinion, it is now time for an integrative approach to subjective influences on ambiguous perception (see Box 2), both in terms of topics and of means of investigation.

Box 2 **Open Questions.**

1. **A common framework for the interpretation of individual differences in patients and in the general population**. The stable determinants of ambiguous perception have been mostly investigated in either pathological samples or in the general population, a striking example in this sense being anxiety defined as a clinical condition or as a personality trait. It remains to be established whether common mechanisms are at play in both cases, possibly investigating the inheritance of ambiguous perception patterns between healthy and affected individuals, and investigating the effects of drugs on ambiguous perception in patients and in the general population.

2. **Individual differences in the voluntary control of ambiguous perception**. The individual differences in bistable perception have been mostly studied in terms of the rate of switching between alternative percepts. However, there is growing evidence that observers have a degree of control on what they perceive and for how long in an ambiguous display. It is largely unknown yet if there are stable individual differences in the ability to voluntarily control the various aspects of bistable perception and whether they correlate with other individual traits.

3. **Cognitive strategies in voluntary control of reversal rate**. The exertion of control on what is currently perceived can be mediated by cognitive strategies or resources whose impact on ambiguous perception has traditionally been studied in isolation: for instance, voluntary control could be achieved via the use of visual imagery strategies. Whereas efforts have been made to understand if and how observers can maintain a specific perceptual alternative in visual awareness (see Section Voluntary Attention), *how* observers control the switching between alternatives is still poorly understood.

4. **Relationship between control of dominance and of reversal rate**. After over a century of studies inquiring the possibility to exert voluntary control on the perception of ambiguous stimuli, the relationship between control of relative dominance and of reversal rate still needs to be specified. Is reversal rate increase attained via the voluntary discharge of an unwanted percept or rather observers are able to bring up to consciousness the desired percept? Likewise, do observers achieve control of relative dominance by voluntarily shortening the dominance period of an unwanted percept or by lengthening the duration of the desired one? What is the relationship between the mechanisms that regulate the control of dominance and of reversal rate? Or is it one and the same mechanism that accounts for both forms of control exertion on ambiguous perception?

5. **Transient factors influencing learning of ambiguous displays**. Learning effects on ambiguous perception likely rely on some form of visual memory and may be influenced by prior history of perceptual dominance. Specifying how different transient aspects may support much longer-lasting perceptual learning effects would help unraveling the functional and neural underpinnings of ambiguous perception.

6. **The role of noise and its relationship with voluntary control of reversal rate**. Much work in the modeling domain has emphasized the role of noise in switching between perceptual alternatives, since adaptation and mutual inhibition between competing neural populations are not enough to explain them on a physiological basis. However, the neurophysiological origin of this noise remains elusive. Does it reflect stochastic processes at the synaptic or neuronal level, or does it reflect an underlying chaotic dynamics of neural populations? Furthermore, is this “noise” modulated during the exertion of cognitive control on what is perceived?

7. **The quest for an overarching computational framework**. While computational models have managed to describe and mechanistically explain a range of fundamental phenomena related to ambiguous perception, it is still unclear to what extent these phenomena might reflect an optimal information processing strategy. To answer this question, future modeling work should attempt to relate successful phenomenological and mechanistic models to normative theories of perception as a form of optimal inference. The resulting framework would be a solid foundation for studying the various transient and stable effects on ambiguous perception.

### Conflict of interest statement

The authors declare that the research was conducted in the absence of any commercial or financial relationships that could be construed as a potential conflict of interest.
